# 2,5,7-Trimethyl-3-(4-methyl­phenyl­sulfon­yl)-1-benzofuran

**DOI:** 10.1107/S160053681202020X

**Published:** 2012-05-16

**Authors:** Hong Dae Choi, Pil Ja Seo, Uk Lee

**Affiliations:** a Department of Chemistry, Dongeui University, San 24 Kaya-dong, Busanjin-gu, Busan 614-714, Republic of Korea; b Department of Chemistry, Pukyong National University, 599-1 Daeyeon 3-dong, Nam-gu, Busan 608-737, Republic of Korea

## Abstract

In the title compound, C_18_H_18_O_3_S, the 4-methyl­phenyl ring makes a dihedral angle of 86.35 (3)° with the mean plane [mean deviation = 0.006 (1) Å] of the benzofuran fragment. In the crystal, mol­ecules are linked by weak C—H⋯O and C—H⋯π inter­actions. The crystal structure also exhibits weak π–π inter­actions between the furan and benzene rings of neighbouring benzofuran systems [centroid–centroid distance = 3.685 (2), inter­planar distance = 3.572 (2) and slippage = 0.906 (2) Å].

## Related literature

For background information and the crystal structures of related compounds, see: Choi *et al.* (2008 ▶, 2010 ▶); Seo *et al.* (2011 ▶).
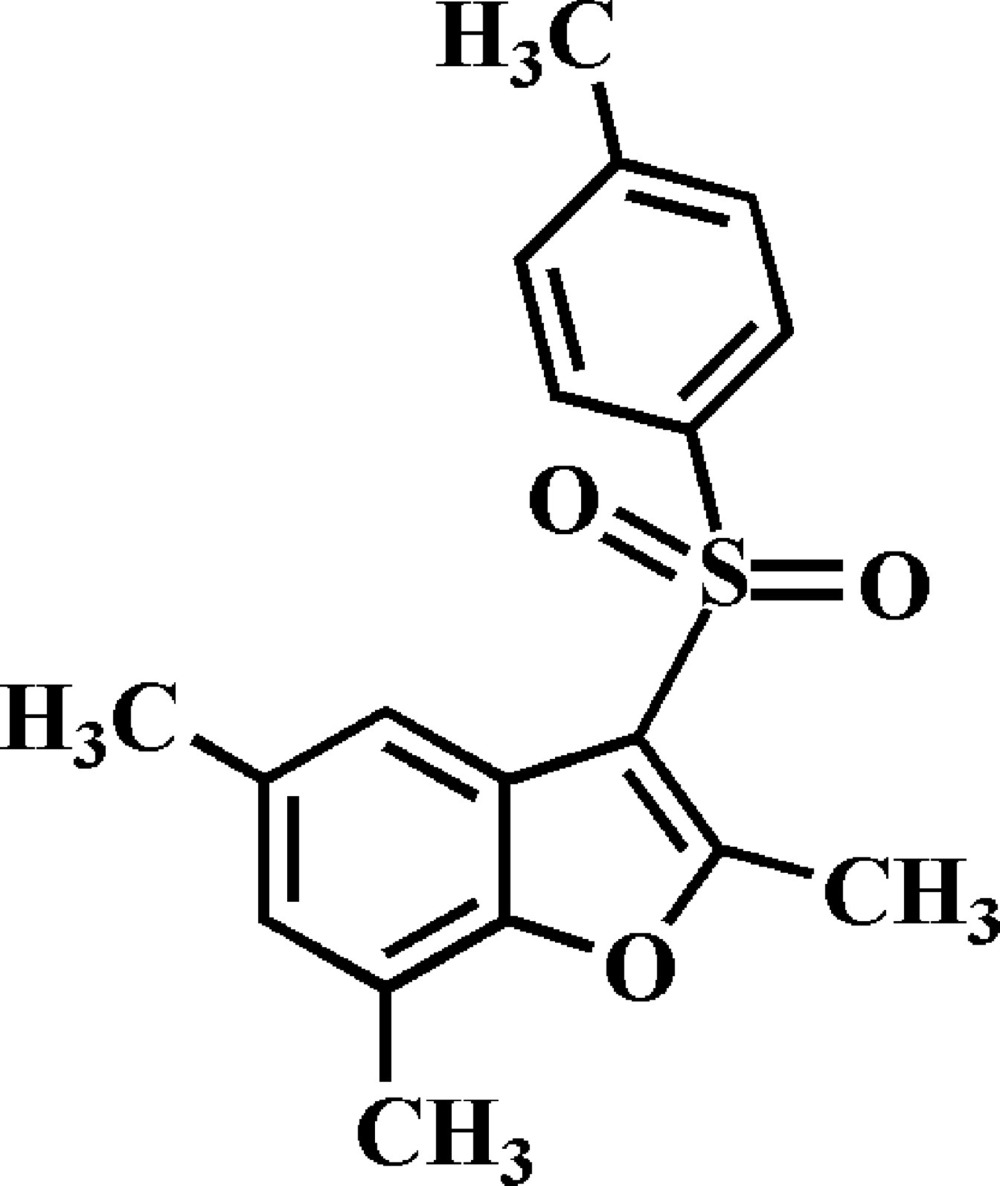


## Experimental

### 

#### Crystal data

C_18_H_18_O_3_S
*M* *_r_* = 314.38
Monoclinic, 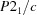

*a* = 9.7666 (2) Å
*b* = 19.4511 (5) Å
*c* = 8.2979 (2) Å
β = 98.541 (1)°
*V* = 1558.88 (6) Å^3^
*Z* = 4
Mo *K*α radiation
μ = 0.22 mm^−1^
*T* = 173 K
0.41 × 0.39 × 0.20 mm

#### Data collection

Bruker SMART APEXII CCD diffractometer
Absorption correction: multi-scan (*SADABS*; Bruker, 2009 ▶) *T*_min_ = 0.916, *T*_max_ = 0.958
15446 measured reflections
3868 independent reflections
3258 reflections with *I* > 2σ(*I*)
*R*_int_ = 0.030

#### Refinement

*R*[*F* ^2^ > 2σ(*F* ^2^)] = 0.042
*wR*(*F* ^2^) = 0.116
*S* = 1.04
3868 reflections
203 parameters
H-atom parameters constrained
Δρ_max_ = 0.30 e Å^−3^
Δρ_min_ = −0.43 e Å^−3^

### 

Data collection: *APEX2* (Bruker, 2009 ▶); cell refinement: *SAINT* (Bruker, 2009 ▶); data reduction: *SAINT*; program(s) used to solve structure: *SHELXS97* (Sheldrick, 2008 ▶); program(s) used to refine structure: *SHELXL97* (Sheldrick, 2008 ▶); molecular graphics: *ORTEP-3* (Farrugia, 1997 ▶) and *DIAMOND* (Brandenburg, 1998 ▶); software used to prepare material for publication: *SHELXL97*.

## Supplementary Material

Crystal structure: contains datablock(s) global, I. DOI: 10.1107/S160053681202020X/sj5234sup1.cif

Structure factors: contains datablock(s) I. DOI: 10.1107/S160053681202020X/sj5234Isup2.hkl

Supplementary material file. DOI: 10.1107/S160053681202020X/sj5234Isup3.cml

Additional supplementary materials:  crystallographic information; 3D view; checkCIF report

## Figures and Tables

**Table 1:** Hydrogen-bond geometry (Å, °) *Cg*1 and *Cg*2 are the centroids of the C12–C17 benzene ring and the C1/C2/C7/O1/C8 furan ring, respectively

*D*—H⋯*A*	*D*—H	H⋯*A*	*D*⋯*A*	*D*—H⋯*A*
C10—H10*C*⋯O3^i^	0.98	2.46	3.310 (2)	146
C9—H9*C*⋯*Cg*1^ii^	0.98	2.93	3.907 (2)	179
C10—H10*B*⋯*Cg*2^iii^	0.98	2.92	3.800 (2)	150

Symmetry codes: (i) 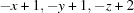; (ii) 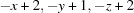; (iii) 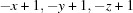.

**Fig. 1.**
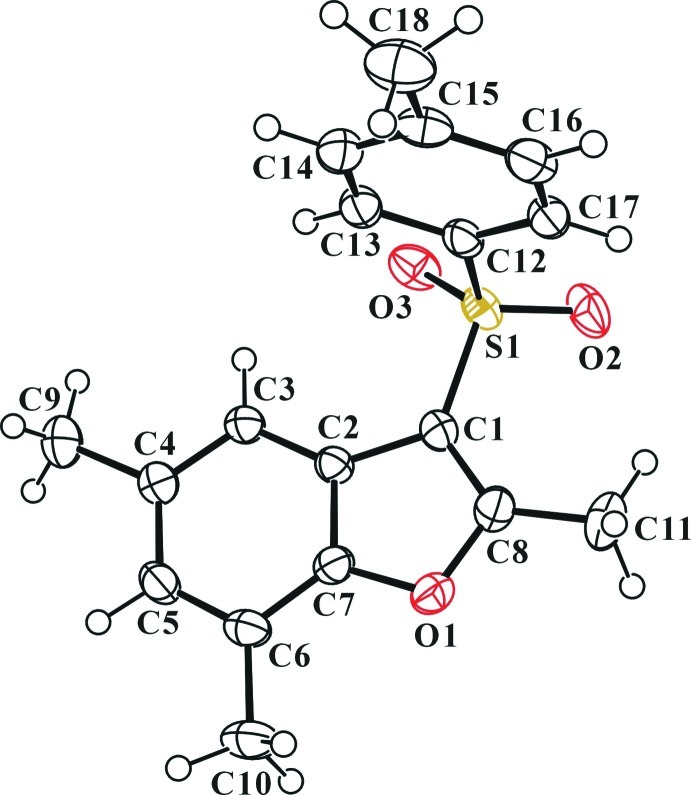
The molecular structure of the title compound with the atom numbering scheme. Displacement ellipsoids are drawn at the 50% probability level. H atoms are presented as small spheres of arbitrary radius.

**Fig. 2.**
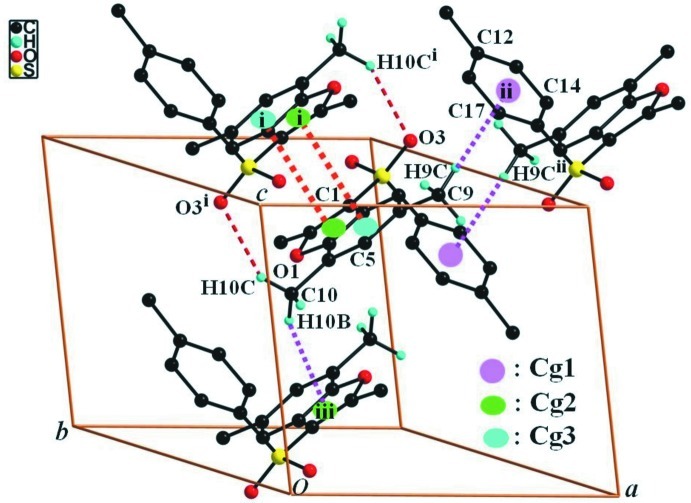
A view of the C—H···O, C—H···π and π···π interactions (dotted lines) in the crystal structure of the title compound. H atoms non-participating in hydrogen-bonding were omitted for clarity. [Symmetry codes: (i) - x + 1, - y + 1, - z + 2; (ii) - x + 2, - y + 1, - z + 2; (iii) - x + 1, - y + 1, - z + 1.]

## supplementary crystallographic information

### Comment

As a part of our ongoing study of 2,5,7-trimethyl-1-benzofuran derivatives containing 3-phenylsulfonyl (Choi *et al.*, 2008), 3-(4-fluorophenylsulfonyl) (Choi *et al.*, 2010) and 3-(3-fluorophenylsulfonyl) (Seo *et al.*, 2011) substituents, we report herein the crystal structure of the title compound.

In the title molecule (Fig. 1), the benzofuran unit is essentially planar, with a mean deviation of 0.006 (1) Å from the least-squares plane defined by the nine constituent atoms. The dihedral angle between the 4-methylphenyl ring and the mean plane of the benzofuran fragment is 86.35 (3)°. In the crystal structure (Fig. 2), molecules are connected by weak intermolecular C—H···O and C—H···π interactions (Table 1). The crystal packing (Fig. 2) also exhibits weak π···π interactions between the furan and benzene rings of neighbouring benzofuran systems, with a Cg2···Cg3^i^ distance of 3.685 (2) Å and an interplanar distance of 3.572 (2) Å resulting in a slippage of 0.906 (2) Å (Cg3 is the centroid of the C2–C7 benzene ring).

### Experimental

3-Chloroperoxybenzoic acid (77%, 560 mg, 2.5 mmol) was added in small portions to a stirred solution of 2,5,7-trimethyl-3-(4-methylphenylsulfanyl)-1-benzofuran (338 mg, 1.2 mmol) in dichloromethane (30 mL) at 273 K. After being stirred at room temperature for 10h, the mixture was washed with saturated sodium bicarbonate solution and the organic layer was separated, dried over magnesium sulfate, filtered and concentrated at reduced pressure. The residue was purified by column chromatography (hexane–ethyl acetate, 4:1 v/v) to afford the title compound as a colorless solid [yield 78%, m.p. 413–414 K; *R*_f_ = 0.48 (hexane–ethyl acetate, 4:1 v/v)]. Single crystals suitable for X-ray diffraction were prepared by slow evaporation of a solution of the title compound in ethyl acetate at room temperature.

### Refinement

All H atoms were positioned geometrically and refined using a riding model, with C–H = 0.95 Å for aryl and 0.98 Å for methyl H atoms. Uiso(H) = 1.2*U*_eq_(C) for aryl and 1.5*U*_eq_(C) for methyl H atoms. The positions of methyl hydrogens were optimized rotationally.

### Crystal data

**Table:**

C_18_H_18_O_3_S	*F*(000) = 664
*M**_r_* = 314.38	*D*_x_ = 1.340 Mg m^−^^3^
Monoclinic, *P*2_1_/*c*	Mo *K*α radiation, λ = 0.71073 Å
Hall symbol: -P 2ybc	Cell parameters from 5168 reflections
*a* = 9.7666 (2) Å	θ = 2.4–28.2°
*b* = 19.4511 (5) Å	µ = 0.22 mm^−^^1^
*c* = 8.2979 (2) Å	*T* = 173 K
β = 98.541 (1)°	Block, colourless
*V* = 1558.88 (6) Å^3^	0.41 × 0.39 × 0.20 mm
*Z* = 4	

### Data collection

**Table:**

Bruker SMART APEXII CCD diffractometer	3868 independent reflections
Radiation source: rotating anode	3258 reflections with *I* > 2σ(*I*)
Graphite multilayer monochromator	*R*_int_ = 0.030
Detector resolution: 10.0 pixels mm^-1^	θ_max_ = 28.4°, θ_min_ = 2.1°
φ and ω scans	*h* = −13→12
Absorption correction: multi-scan (*SADABS*; Bruker, 2009)	*k* = −21→25
*T*_min_ = 0.916, *T*_max_ = 0.958	*l* = −11→10
15446 measured reflections	

### Refinement

**Table:**

Refinement on *F*^2^	Primary atom site location: structure-invariant direct methods
Least-squares matrix: full	Secondary atom site location: difference Fourier map
*R*[*F*^2^ > 2σ(*F*^2^)] = 0.042	Hydrogen site location: difference Fourier map
*wR*(*F*^2^) = 0.116	H-atom parameters constrained
*S* = 1.04	*w* = 1/[σ^2^(*F*_o_^2^) + (0.0582*P*)^2^ + 0.5651*P*] where *P* = (*F*_o_^2^ + 2*F*_c_^2^)/3
3868 reflections	(Δ/σ)_max_ = 0.001
203 parameters	Δρ_max_ = 0.30 e Å^−^^3^
0 restraints	Δρ_min_ = −0.43 e Å^−^^3^

### Special details

**Table:**

Geometry. All esds (except the esd in the dihedral angle between two l.s. planes) are estimated using the full covariance matrix. The cell esds are taken into account individually in the estimation of esds in distances, angles and torsion angles; correlations between esds in cell parameters are only used when they are defined by crystal symmetry. An approximate (isotropic) treatment of cell esds is used for estimating esds involving l.s. planes.
Refinement. Refinement of F^2^ against ALL reflections. The weighted R-factor wR and goodness of fit S are based on F^2^, conventional R-factors R are based on F, with F set to zero for negative F^2^. The threshold expression of F^2^ > 2sigma(F^2^) is used only for calculating R-factors(gt) etc. and is not relevant to the choice of reflections for refinement. R-factors based on F^2^ are statistically about twice as large as those based on F, and R- factors based on ALL data will be even larger.

### Fractional atomic coordinates and isotropic or equivalent isotropic displacement parameters (Å^2^)

**Table:**

	*x*	*y*	*z*	*U*_iso_*/*U*_eq_	
S1	0.77821 (4)	0.617621 (19)	0.95912 (5)	0.03124 (12)	
O1	0.44316 (10)	0.54239 (6)	0.69953 (13)	0.0335 (3)	
O2	0.73376 (14)	0.68604 (6)	0.99116 (16)	0.0445 (3)	
O3	0.84855 (13)	0.57685 (6)	1.09059 (14)	0.0401 (3)	
C1	0.63766 (15)	0.56994 (7)	0.86672 (18)	0.0281 (3)	
C2	0.62873 (14)	0.49571 (7)	0.86149 (17)	0.0259 (3)	
C3	0.70807 (15)	0.44093 (8)	0.93316 (18)	0.0295 (3)	
H3	0.7907	0.4490	1.0067	0.035*	
C4	0.66347 (16)	0.37470 (8)	0.8946 (2)	0.0328 (3)	
C5	0.54034 (16)	0.36373 (8)	0.7862 (2)	0.0342 (3)	
H5	0.5122	0.3177	0.7612	0.041*	
C6	0.45823 (15)	0.41655 (9)	0.71436 (19)	0.0321 (3)	
C7	0.50737 (14)	0.48180 (8)	0.75612 (18)	0.0276 (3)	
C8	0.52485 (16)	0.59479 (8)	0.7677 (2)	0.0329 (3)	
C9	0.7482 (2)	0.31384 (9)	0.9655 (3)	0.0463 (4)	
H9A	0.7937	0.2924	0.8806	0.069*	
H9B	0.6873	0.2803	1.0071	0.069*	
H9C	0.8185	0.3294	1.0547	0.069*	
C10	0.32615 (17)	0.40458 (11)	0.5996 (2)	0.0438 (4)	
H10A	0.3180	0.3557	0.5713	0.066*	
H10B	0.3273	0.4318	0.5003	0.066*	
H10C	0.2471	0.4185	0.6522	0.066*	
C11	0.4770 (2)	0.66466 (10)	0.7171 (3)	0.0498 (5)	
H11A	0.4870	0.6718	0.6026	0.075*	
H11B	0.5328	0.6987	0.7849	0.075*	
H11C	0.3795	0.6698	0.7305	0.075*	
C12	0.88605 (15)	0.62242 (7)	0.80711 (18)	0.0281 (3)	
C15	1.04793 (16)	0.62512 (9)	0.55823 (19)	0.0355 (4)	
C16	0.97063 (16)	0.68243 (9)	0.5909 (2)	0.0360 (4)	
H16	0.9738	0.7228	0.5273	0.043*	
C17	0.88939 (16)	0.68161 (8)	0.71428 (19)	0.0323 (3)	
H17	0.8369	0.7209	0.7350	0.039*	
C18	1.1300 (2)	0.62519 (11)	0.4184 (2)	0.0504 (5)	
H18A	1.0714	0.6089	0.3196	0.076*	
H18B	1.2102	0.5948	0.4439	0.076*	
H18C	1.1617	0.6720	0.4006	0.076*	
C13	0.96346 (15)	0.56489 (8)	0.77825 (19)	0.0317 (3)	
H13	0.9609	0.5247	0.8426	0.038*	
C14	1.04408 (16)	0.56690 (8)	0.6550 (2)	0.0355 (3)	
H14	1.0979	0.5279	0.6359	0.043*	

### Atomic displacement parameters (Å^2^)

**Table:**

	*U*^11^	*U*^22^	*U*^33^	*U*^12^	*U*^13^	*U*^23^
S1	0.0373 (2)	0.0257 (2)	0.0299 (2)	−0.00632 (14)	0.00250 (15)	−0.00488 (14)
O1	0.0277 (5)	0.0347 (6)	0.0360 (6)	0.0051 (4)	−0.0020 (4)	−0.0004 (5)
O2	0.0568 (7)	0.0282 (6)	0.0508 (7)	−0.0068 (5)	0.0156 (6)	−0.0142 (5)
O3	0.0457 (6)	0.0439 (7)	0.0279 (6)	−0.0111 (5)	−0.0041 (5)	0.0011 (5)
C1	0.0300 (7)	0.0248 (7)	0.0295 (7)	−0.0007 (5)	0.0039 (6)	−0.0024 (6)
C2	0.0259 (6)	0.0257 (7)	0.0256 (7)	−0.0019 (5)	0.0022 (5)	−0.0022 (5)
C3	0.0268 (6)	0.0281 (7)	0.0321 (8)	−0.0014 (5)	−0.0005 (5)	0.0012 (6)
C4	0.0333 (7)	0.0275 (8)	0.0373 (8)	−0.0011 (6)	0.0046 (6)	0.0016 (6)
C5	0.0350 (8)	0.0270 (7)	0.0410 (9)	−0.0067 (6)	0.0072 (6)	−0.0072 (6)
C6	0.0263 (7)	0.0385 (9)	0.0314 (8)	−0.0060 (6)	0.0040 (6)	−0.0091 (6)
C7	0.0242 (6)	0.0306 (7)	0.0273 (7)	0.0022 (5)	0.0016 (5)	−0.0013 (6)
C8	0.0334 (7)	0.0299 (8)	0.0355 (8)	0.0045 (6)	0.0051 (6)	−0.0001 (6)
C9	0.0503 (10)	0.0282 (9)	0.0590 (12)	0.0037 (7)	0.0032 (8)	0.0053 (8)
C10	0.0293 (7)	0.0596 (11)	0.0405 (9)	−0.0081 (7)	−0.0009 (7)	−0.0155 (8)
C11	0.0514 (10)	0.0350 (9)	0.0611 (12)	0.0153 (8)	0.0022 (9)	0.0067 (9)
C12	0.0283 (7)	0.0251 (7)	0.0287 (7)	−0.0043 (5)	−0.0030 (5)	−0.0012 (6)
C15	0.0278 (7)	0.0457 (9)	0.0311 (8)	−0.0095 (6)	−0.0021 (6)	−0.0032 (7)
C16	0.0346 (8)	0.0359 (8)	0.0349 (8)	−0.0085 (6)	−0.0028 (6)	0.0068 (7)
C17	0.0312 (7)	0.0262 (7)	0.0375 (8)	−0.0023 (6)	−0.0018 (6)	0.0019 (6)
C18	0.0444 (10)	0.0665 (13)	0.0418 (10)	−0.0132 (9)	0.0108 (8)	−0.0055 (9)
C13	0.0313 (7)	0.0272 (7)	0.0342 (8)	−0.0014 (5)	−0.0033 (6)	0.0018 (6)
C14	0.0305 (7)	0.0343 (8)	0.0400 (9)	0.0008 (6)	−0.0008 (6)	−0.0046 (7)

### Geometric parameters (Å, º)

**Table:**

S1—O2	1.4369 (12)	C9—H9C	0.9800
S1—O3	1.4382 (12)	C10—H10A	0.9800
S1—C1	1.7377 (15)	C10—H10B	0.9800
S1—C12	1.7617 (16)	C10—H10C	0.9800
O1—C8	1.3643 (19)	C11—H11A	0.9800
O1—C7	1.3840 (17)	C11—H11B	0.9800
C1—C8	1.361 (2)	C11—H11C	0.9800
C1—C2	1.447 (2)	C12—C17	1.388 (2)
C2—C7	1.3909 (19)	C12—C13	1.391 (2)
C2—C3	1.397 (2)	C15—C14	1.392 (2)
C3—C4	1.382 (2)	C15—C16	1.395 (2)
C3—H3	0.9500	C15—C18	1.505 (2)
C4—C5	1.407 (2)	C16—C17	1.385 (2)
C4—C9	1.513 (2)	C16—H16	0.9500
C5—C6	1.383 (2)	C17—H17	0.9500
C5—H5	0.9500	C18—H18A	0.9800
C6—C7	1.383 (2)	C18—H18B	0.9800
C6—C10	1.504 (2)	C18—H18C	0.9800
C8—C11	1.478 (2)	C13—C14	1.381 (2)
C9—H9A	0.9800	C13—H13	0.9500
C9—H9B	0.9800	C14—H14	0.9500
			
O2—S1—O3	119.59 (8)	C6—C10—H10A	109.5
O2—S1—C1	109.59 (7)	C6—C10—H10B	109.5
O3—S1—C1	107.30 (7)	H10A—C10—H10B	109.5
O2—S1—C12	108.21 (7)	C6—C10—H10C	109.5
O3—S1—C12	107.66 (7)	H10A—C10—H10C	109.5
C1—S1—C12	103.27 (7)	H10B—C10—H10C	109.5
C8—O1—C7	106.73 (11)	C8—C11—H11A	109.5
C8—C1—C2	107.24 (13)	C8—C11—H11B	109.5
C8—C1—S1	126.32 (12)	H11A—C11—H11B	109.5
C2—C1—S1	125.87 (11)	C8—C11—H11C	109.5
C7—C2—C3	119.08 (13)	H11A—C11—H11C	109.5
C7—C2—C1	104.77 (12)	H11B—C11—H11C	109.5
C3—C2—C1	136.15 (13)	C17—C12—C13	120.90 (15)
C4—C3—C2	118.45 (13)	C17—C12—S1	120.47 (12)
C4—C3—H3	120.8	C13—C12—S1	118.59 (11)
C2—C3—H3	120.8	C14—C15—C16	118.43 (15)
C3—C4—C5	119.97 (14)	C14—C15—C18	120.57 (16)
C3—C4—C9	120.27 (15)	C16—C15—C18	120.97 (16)
C5—C4—C9	119.75 (15)	C17—C16—C15	121.36 (15)
C6—C5—C4	123.28 (14)	C17—C16—H16	119.3
C6—C5—H5	118.4	C15—C16—H16	119.3
C4—C5—H5	118.4	C16—C17—C12	118.87 (15)
C7—C6—C5	114.61 (13)	C16—C17—H17	120.6
C7—C6—C10	122.29 (16)	C12—C17—H17	120.6
C5—C6—C10	123.10 (15)	C15—C18—H18A	109.5
C6—C7—O1	125.01 (13)	C15—C18—H18B	109.5
C6—C7—C2	124.59 (14)	H18A—C18—H18B	109.5
O1—C7—C2	110.40 (13)	C15—C18—H18C	109.5
C1—C8—O1	110.86 (13)	H18A—C18—H18C	109.5
C1—C8—C11	133.75 (16)	H18B—C18—H18C	109.5
O1—C8—C11	115.37 (14)	C14—C13—C12	119.27 (14)
C4—C9—H9A	109.5	C14—C13—H13	120.4
C4—C9—H9B	109.5	C12—C13—H13	120.4
H9A—C9—H9B	109.5	C13—C14—C15	121.17 (15)
C4—C9—H9C	109.5	C13—C14—H14	119.4
H9A—C9—H9C	109.5	C15—C14—H14	119.4
H9B—C9—H9C	109.5		
			
O2—S1—C1—C8	30.29 (16)	C1—C2—C7—C6	179.59 (14)
O3—S1—C1—C8	161.58 (14)	C3—C2—C7—O1	179.33 (13)
C12—S1—C1—C8	−84.84 (15)	C1—C2—C7—O1	−0.47 (16)
O2—S1—C1—C2	−159.46 (13)	C2—C1—C8—O1	0.35 (17)
O3—S1—C1—C2	−28.17 (15)	S1—C1—C8—O1	172.09 (11)
C12—S1—C1—C2	85.40 (14)	C2—C1—C8—C11	−177.50 (19)
C8—C1—C2—C7	0.08 (16)	S1—C1—C8—C11	−5.8 (3)
S1—C1—C2—C7	−171.71 (11)	C7—O1—C8—C1	−0.64 (17)
C8—C1—C2—C3	−179.68 (17)	C7—O1—C8—C11	177.64 (14)
S1—C1—C2—C3	8.5 (3)	O2—S1—C12—C17	−14.89 (14)
C7—C2—C3—C4	0.8 (2)	O3—S1—C12—C17	−145.45 (12)
C1—C2—C3—C4	−179.43 (16)	C1—S1—C12—C17	101.24 (13)
C2—C3—C4—C5	−0.4 (2)	O2—S1—C12—C13	167.49 (12)
C2—C3—C4—C9	178.24 (15)	O3—S1—C12—C13	36.93 (13)
C3—C4—C5—C6	−0.4 (3)	C1—S1—C12—C13	−76.38 (13)
C9—C4—C5—C6	−179.01 (16)	C14—C15—C16—C17	−1.3 (2)
C4—C5—C6—C7	0.6 (2)	C18—C15—C16—C17	176.77 (15)
C4—C5—C6—C10	−179.54 (16)	C15—C16—C17—C12	0.3 (2)
C5—C6—C7—O1	179.95 (14)	C13—C12—C17—C16	0.5 (2)
C10—C6—C7—O1	0.1 (2)	S1—C12—C17—C16	−177.10 (11)
C5—C6—C7—C2	−0.1 (2)	C17—C12—C13—C14	−0.3 (2)
C10—C6—C7—C2	−179.97 (15)	S1—C12—C13—C14	177.35 (11)
C8—O1—C7—C6	−179.37 (15)	C12—C13—C14—C15	−0.7 (2)
C8—O1—C7—C2	0.69 (16)	C16—C15—C14—C13	1.5 (2)
C3—C2—C7—C6	−0.6 (2)	C18—C15—C14—C13	−176.57 (14)

### Hydrogen-bond geometry (Å, º)

Cg1 and Cg2 are the centroids of the C12–C17 benzene ring and the C1/C2/C7/O1/C8 furan ring, respectively

**Table:**

*D*—H···*A*	*D*—H	H···*A*	*D*···*A*	*D*—H···*A*
C10—H10*C*···O3^i^	0.98	2.46	3.310 (2)	146
C9—H9*C*···*Cg*1^ii^	0.98	2.93	3.907 (2)	179
C10—H10*B*···*Cg*2^iii^	0.98	2.92	3.800 (2)	150

Symmetry codes: (i) −*x*+1, −*y*+1, −*z*+2; (ii) −*x*+2, −*y*+1, −*z*+2; (iii) −*x*+1, −*y*+1, −*z*+1.
